# Foliar Application of Cerium Oxide-Salicylic Acid Nanoparticles (CeO_2_:SA Nanoparticles) Influences the Growth and Physiological Responses of *Portulaca oleracea* L. under Salinity

**DOI:** 10.3390/ijms23095093

**Published:** 2022-05-03

**Authors:** Mohammad Bagher Hassanpouraghdam, Lamia Vojodi Mehrabani, Zahra Bonabian, Mohammad Ali Aazami, Farzad Rasouli, Marcin Feldo, Maciej Strzemski, Sławomir Dresler

**Affiliations:** 1 Department of Horticultural Science, Faculty of Agriculture, University of Maragheh, Maragheh 55181-83111, Iran; aazami58@gmail.com (M.A.A.); farrasoli@gmail.com (F.R.); 2 Department of Agronomy and Plant Breeding, Azarbaijan Shahid Madani University, Tabriz 53751-71379, Iran; vojodilamia@gmail.com (L.V.M.); bonabizahra7@gmail.com (Z.B.); 3 Department of Vascular Surgery, Medical University of Lublin, 11 Staszica St., 20-081 Lublin, Poland; martinf@interia.pl; 4 Department of Analytical Chemistry, Medical University of Lublin, 20-093 Lublin, Poland; maciej.strzemski@poczta.onet.pl (M.S.); slawomir.dresler@umlub.pl (S.D.); 5 Department of Plant Physiology and Biophysics, Institute of Biological Science, Maria Curie-Skłodowska University, Akademicka 19, 20-033 Lublin, Poland

**Keywords:** *Portulaca oleracea*, elemental content, malondialdehyde, phenolic compound

## Abstract

In the present study, the effects of foliar application of salicylic acid (100 μM), cerium oxide (50 mg L^−1^), and cerium oxide:salicylic acid nanoparticles (CeO_2_: SA-nanoparticles, 50 mg L^−1^ + 100 μM) on the growth and physiological responses of purslane (*Portulaca oleracea* L.) were examined in non-saline and saline conditions (50 and 100 mM NaCl salinity). Foliar applications mitigated salinity-induced adverse effects, and the highest plant height and N, P, Mg, and Mn content were recorded in the variant with non-saline × foliar use of CeO_2_: SA-nanoparticles. The highest values of fresh and dry weight were noted in the treatment with no-salinity × foliar use of CeO_2_:SA-nanoparticles. The highest number of sub-branches was observed in the foliar treatments with CeO_2_-nanoparticles and CeO_2_:SA-nanoparticles without salinity stress, while the lowest number was noted in the 100 mM NaCl treatment. Moreover, the foliar application of CeO_2_:SA-nanoparticles and cerium-oxide nanoparticles improved the total soluble solid content, K, Fe, Zn, Ca, chlorophyll a, and oil yield in the plants. The salinity of 0 and 50 mM increased the K content, 1000-seed weight, total soluble solid content, and chlorophyll b content. The use of 100 mM NaCl with no-foliar spray increased the malondialdehyde, Na, and H_2_O_2_ content and the Na^+^/K^+^ ratio. No-salinity and 50 mM NaCl × CeO_2_: SA-nanoparticle interactions improved the anthocyanin content in plants. The phenolic content was influenced by NaCl_100_ and the foliar use of CeO_2_:SA-nanoparticles. The study revealed that the foliar treatment with CeO_2_:SA-nanoparticles alleviated the side effects of salinity by improving the physiological responses and growth-related traits of purslane plants.

## 1. Introduction

Purslane (*Portulaca oleracea*) is an annual succulent plant from the family Portulacaceae. It contains several antioxidant compounds, proteins, vitamins, minerals, polysaccharides, alkaloids, phenolics, flavonoids, and omega-3 fatty acids with many applications in the food and pharmaceutical industries. In traditional medicine, purslane has been used as a febrifuge, disinfectant, anti-bacterial, anti-inflammatory, and wound healing plant [1]. The rapid population growth and the great need for plant material have imposed enormous pressures on ecological systems. The collection from natural habitats never meets the ever-growing needs of several industries. Therefore, it is necessary to make emergent decisions for the large-scale production of medicinal plants in sustainable agricultural production systems [2].

The Food and Agriculture Organization (FAO) estimates that there are more than 833 million hectares of salt-affected soils around the globe (8.7% of the planet). Most of them can be found in naturally arid or semi-arid environments in Africa, Asia, and Latin America. Moreover, 20 to 50 percent of irrigated soils in all continents are excessively salty, which implies that over 1.5 billion people worldwide face significant challenges in growing food due to soil degradation [3]. In Iran, more than 50% of agricultural soil is in the face of salinity exposure [3]. Salinity impacts plant morphological and physiological characteristics, growth, and productivity by imposing ionic toxicity, ionic imbalances, reduced K^+^ absorption, and osmotic and later oxidative stress [4,5]. Salinity induces diverse nutritional disorders in plants via interference in the nutrient uptake and ion competition, translocation, and partitioning toward the growing sites inside plants [6,7]. The decreased potassium uptake triggers water deficiency in the plant resulting in oxidative damage to proteins and fats and disruption of normal cell metabolism [8]. Closure of stomata due to salinity stress reduces NADPH consumption in the Calvin cycle. The generated free radicals reduce the use of NADPH in chemical reactions, thereby disrupting several physiological processes. In salinity stress conditions, the accumulation of soluble sugars increases in favor of adaptation to the salinity stress conditions. The declined water potential and photosynthesis under salinity stress reduce and even stop plant growth, development, and productivity [4,5,7,8]. The outcome of all the interferences mentioned is the oxidative damage to proteins and lipids and the chaos in the normal cell metabolism [5].

Antioxidant enzymes and non-enzymatic antioxidant compounds have prominent roles in ameliorating the side effects of ROS radicals [9]. Excessive accumulation of ROS causes severe damage to cell membrane phospholipids, leading to lipid peroxidation, ion leakage, and reduced plant yield. Plants are equipped with enzymatic and non-enzymatic defense systems to eliminate or reduce ROS production. Foliar application of low-molecular-weight water-soluble substances (osmolytes such as polyamines, proline, and soluble solids) is an efficient strategy to combat stress in plants. The presence of enzymes and antioxidant compounds plays a vital role in reducing the damage caused by oxygen free radicals produced in the cell [9]. Salinity stress causes various nutritional abnormalities in the plant, which may be related to the adverse effects of salinity on the ability to absorb, transport, and distribute nutrients [4,9]. The overall result is oxidative damage to proteins and fats and disruption of normal cell metabolism [5]. In purslane, the concentration of NO_3_^-^ and soluble protein, Rubisco, ascorbic acid, and phenolic content were increased under 100 mM salinity. The highest Mg, Ca, and K content was recorded in no-salinity conditions. With salinity up to 300 mM, the leaf area index, water content, cytb_6_f concentration, and net photosynthesis potential declined markedly. However, the proline content was improved. This indicates that the plant can withstand salinity of up to 100 mM [10].

In the last decade, the decline in agricultural production due to the enormous environmental changes has been a massive challenge for food security in many countries, especially those with more progressive climate changes. Therefore, it is essential to find a way to accelerate the plant adaptation process to environmental changes. With advances in the multidisciplinary science domains, nanotechnology has emerged as one of the most influential and effective tools in combating environmental stress factors in plants. Nanoparticles (due to their high specific surface area, shape, and size) play an essential role in physiological processes, photosynthesis, and the activity of antioxidant enzymes in plants [7].

Due to their unique electrical, optical, and thermal properties, cerium oxide (lanthanide) nanoparticles have several industrial, biological, therapeutic, and agricultural applications [11]. The widespread use of nanoparticles in various industries has raised concerns about the health of living organisms and humans due to the environmental conditions associated with these materials [11]. The continuous or repeated exposure (pharmaceutical or ecological) to high doses of cerium oxide nanoparticles disrupts cell wall membranes in *Cyanobacteria* species [12], damages the human lung [13], and harms the liver and spleen tissues in mice [14]. Since cerium oxide can remain in internal organs such as the spleen, liver, and bone marrow for a long time, it is necessary to consider the effect of these substances on human health. In a study on the impact of cerium oxide nanoparticles on reproductive organs and the systematic function of internal organs of rats, the application of an appropriate concentration (125 µg mL^−1^) had no side effects on the health of these organs [11]. Another study found that cerium oxide protected human cells against oxidative stress and inflammation by eliminating the toxic effects of ROS due to the redox reaction of Ce^3+^/Ce^4+^ [15]. Cerium oxide also stimulates the production of SOD, which acts as a powerful antioxidant and thus helps living organisms to survive under stress [15]. In a study on coriander, 125 mg kg^−1^ of cerium oxide in the soil increased root length, improved plant growth, and enhanced catalase activity in the plant. However, 500 mg kg^−1^ cerium oxide application resulted in the accumulation of cerium oxide in plant tissues, which may be harmful to human health [16].

However, more in-depth studies should be conducted to confirm the possible human health side-effects of foliar application of small amounts of cerium oxide treatments on edible plants in stress conditions.

In agriculture, cerium oxide can eliminate ROS in stress conditions (light, temperature, salinity, drought, etc.). It maintains ionic homeostasis, chlorophyll biosynthesis, and the potassium-to-sodium ratio in the plant. The effect of cerium oxide nanoparticles depends on the plant species, growth conditions, concentration, and duration of plant exposure to the stress [7,17,18]. The results of studies performed on cotton [18] under salinity stress and foliar application of cerium oxide nanoparticles showed that the foliar treatment had a positive effect on reducing salinity stress, increased chlorophyll content and plant biomass, and decreased malondialdehyde and hydrogen peroxide content in the plant.

Salicylic acid is a phenolic compound that plays an essential role in regulating physiological and biochemical activities in plants. Salicylic acid mediates plant growth and development, flower induction, nutrient uptake and transport, pigment biosynthesis, cell respiration, cell membrane stability, and stomatal movement and enhances the antioxidant defense system [19,20,21]. The effect of salicylic acid on the plant depends on the plant species, its developmental stage, the concentration of utilized salicylic acid, and environmental conditions [19]. A study on sage showed that salinity reduced yields, but treatment with salicylic acid increased plant tolerance to stress [19]. Similar results have been reported on the reduction in salinity stress through the application of salicylic acid in rosemary [22]. In studies performed on *Trachyspormum ammi* L., it was found that salinity stress (8–12 dS/m) added up the content of sodium, MDA, proline, and the activity of antioxidant enzymes. At the same time, potassium, iron, leaf water content, photosynthetic pigment content, and grain yield were reduced [23]. The application of salicylic acid and iron nanoparticles increased potassium uptake, K/Na ratio, iron content, and activity of antioxidant enzymes (CAT and SOD) in the plant [23].

This study aimed to evaluate the effects of foliar application of salicylic acid, cerium oxide nanoparticles, and CeO_2_:SA-nanoparticles on the yield (fresh and dry weight of the plant and 1000-seed weight), physiological responses, and elemental content in *Portulaca oleracea* under salinity stress.

## 2. Results

### 2.1. Fresh and Dry Weight (Biomass)

The salinity stress × foliar applications had a significant effect on the fresh and dry weight of purslane (Table 1). A significantly reduced amount of fresh and dry weight was shown in response to the salinity stress, whereas the foliar application of salicylic acid, CeO_2_-nanoparticles, and CeO_2_:SA-nanoparticles improved the traits. The highest fresh and dry weights were obtained in the plants subjected to the CeO_2_:SA-nanoparticles foliar application, which increased by 142% and 151% compared to the control, respectively. However, 100 mM of NaCl diminished the dry weight up to 98% compared to the control (Table 2).

### 2.2. Root Fresh and Dry Weight

The findings showed that the foliar application affected the fresh and dry weight of roots under the salinity stress (Table 1). Although the salinity stress reduced root fresh and dry weight, salicylic acid, CeO_2_-nanoparticles, and CeO_2_:SA-nanoparticles recuperated the traits. The highest and the lowest root fresh and dry weight values were observed in the foliar application of CeO_2_:SA-nanoparticles without salinity stress and 100 mM of NaCl without any foliar treatments. The fresh and dry weights of roots in the plants subjected to CeO_2_:SA-nanoparticles exhibited a 3.79- and 9-fold increase, respectively (Table 2).

### 2.3. Plant Height and Number of Branches

Plant height (P ≤ 1%) and the number of sub-branches (P ≤ 1%) were significantly affected by the foliar application of salicylic acid, cerium oxide, and CeO_2_:SA-nanoparticles under salinity stress (Table 1). The salinity stress considerably reduced the plant height and the number of branches, but the foliar application of salicylic acid, CeO_2_-nanoparticles, and CeO_2_:SA-nanoparticles mitigated the salinity effects. The highest and the lowest plant height and number of sub-branches were observed in the purslane supplemented with CeO_2_:SA-nanoparticles and under 100 mM of NaCl, respectively. The results showed that CeO_2_:SA-nanoparticles improved these traits in the purslane plants more efficiently than salicylic acid and CeO_2_-NPs under the salinity stress (Table 2).

### 2.4. 1000-Seed Weight

The foliar applications and salinity stress significantly influenced the 1000-seed weight (P ≤ 1%) (Table 1). The salinity stress decreased the 1000-seed weight, such that the highest weight was observed in the control plants and the lowest was recorded in the 50 mM salinity variant. The 1000-seed weight was reduced by 25% in the plants exposed to 100 mM NaCl, compared to the control (Table 3). The foliar application of salicylic acid, CeO_2_-nanoparticles, and CeO_2_:SA-nanoparticles increased the 1000-seed weight of the plant. The highest 1000-seed weight was noted in the variant of the foliar application of CeO_2_:SA-nanoparticles, which was 27% higher than in the control (Table 4).

### 2.5. TSS Content

TSS was significantly affected by the independent effects of the experimental treatments (P ≤ 1%) (Table 1). The TSS content was raised by 31% in the purslane plants under 100 mM of NaCl, compared to control (Table 3). The foliar application of CeO_2_:SA-nanoparticles improved the TSS content, compared to the control (Table 4).

### 2.6. Oil Yield

The salinity stress and foliar application of salicylic acid, CeO_2_-nanoparticles, and CeO_2_:SA-nanoparticles significantly affected the purslane oil yield (Table 1). The salinity of 100 mM caused a 42% reduction in oil yield compared to the control, which exhibited the highest oil yield (Table 3). All of the foliar applications improved the oil yield compared to the control. The highest oil yield was recorded in the purslane plants supplemented with CeO_2_:SA-nanoparticles (161% higher than in the control) (Table 4).

### 2.7. Chlorophyll a and b Content

The chlorophyll a and b contents were significantly affected by the use of NaCl and the foliar applications (P ≤ 1%) (Table 5). The salinity stress reduced their content. On the other hand, salicylic acid, CeO_2_-nanoparticles, and CeO_2_:SA-nanoparticles increased their levels. The 100 mM NaCl salinity stress reduced the chlorophyll a and b content by 79% and 57%, respectively, compared to the control. At the same time, CeO_2_:SA-nanoparticles improved these traits by 80% and 49% compared to the control (Table 3 and Table 4).

### 2.8. Total Phenolic, Flavonoid, and Anthocyanin content

The salinity stress with salicylic acid, CeO_2_-nanoparticles, and CeO_2_:SA-nanoparticles had a significant impact on the content of phenolics (P ≤ 1%), anthocyanins (P ≤ 5%), and flavonoids (P ≤ 1%) (Table 5). The foliar spray with salicylic acid, CeO_2_-nanoparticles, and CeO_2_:SA-nanoparticles increased the total phenolic, total flavonoid, and anthocyanin content in purslane along with salinity conditions. The highest values were obtained in the foliar treatment with 100 mM NaCl × CeO_2_:SA-nanoparticles, and the lowest content of these compounds was recorded in the control (Table 6).

### 2.9. Malondialdehyde Content

The results showed that the salinity stress enhanced the MDA content, but the foliar application significantly reduced this parameter (P ≤ 1%) (Table 7). The highest MDA content, which increased by 65% compared to the control, was observed in the 100 mM NaCl variant without foliar application. The foliar application of CeO_2_-nanoparticles and CeO_2_:SA-nanoparticles reduced the MDA content more potently than salicylic acid under the salinity stress (Table 6).

### 2.10. Hydrogen Peroxide Content

The H_2_O_2_ content in purslane was affected by the salinity stress and the foliar use of salicylic acid, CeO_2_-nanoparticles, and CeO_2_:SA-nanoparticles (P ≤ 1%) (Table 7). The H_2_O_2_ content increased in plants under the salinity stress but declined in the foliar application variant (Table 6).

### 2.11. Catalase Activity

The NaCl treatment and foliar application (P ≤ 5%) significantly influenced catalase activity in purslane (Table 7). The salinity stress increased catalase activity. Moreover, salicylic acid, CeO_2_-nanoparticles, and CeO_2_:SA-nanoparticles enhanced catalase activity in plants subjected to 50 mM NaCl more efficiently than in the 100 mM treatment. The highest catalase activity was observed in the treatment with 50 mM salinity and CeO_2_-nanoparticles, and the lowest catalase activity was recorded in the control plants. However, CeO_2_-nanoparticles × 50 mM salinity stress produced a 2-fold increase in catalase activity compared to the control. A decrease in catalase activity was observed at 100 mM salinity stress with all foliar applications (Table 6).

### 2.12. Proline Content

The proline content in the purslane plants was considerably affected by the foliar applications under the salinity stress (Table 7). An enhancement of the proline content was induced by the salinity stress, while it was reduced in the plants subjected to the foliar applications. The highest and the lowest proline content was determined in the 100 mM of NaCl treatment and the control, respectively (Table 6).

### 2.13. Nitrogen Content

All foliar applications with salinity treatments (0, 50, and 100 mM) increased the N content in purslane compared to the control, but the highest nitrogen content was obtained in the plants sprayed with CeO_2_:SA-nanoparticles, which showed a 255% increase compared to the control.

### 2.14. Phosphorus Content

Foliar application of salicylic acid and cerium oxide increased the phosphorus content at all levels of salinity stress compared to the control. The highest phosphorus content was observed in the purslane plants exposed to CeO_2_:SA-nanoparticles, which showed a 104% increase compared to the control. The lowest phosphorus content was recorded in the 100 mM NaCl treatment without foliar application, in which it was decreased by 58% compared to the control.

### 2.15. Potassium and Calcium Content

The potassium and calcium content was significantly affected by the salinity stress and foliar applications (P ≤ 1%) (Table 8). The salinity stress reduced the potassium content, and its highest and lowest levels were determined in the control and the 100 mM NaCl treatment, respectively (Table 9). The foliar applications improved the potassium content, and the highest and the lowest values were obtained in the plants subjected to the foliar treatments with CeO_2_:SA-nanoparticles and in the control, respectively (Table 10).

The salinity stress decreased the calcium content. The treatment with 100 mM NaCl reduced its value by 53% compared to the control (Table 9). In turn, all foliar applications improved the calcium content. However, the highest calcium content was observed in the foliar application of CeO_2_-nanoparticles and CeO_2_:SA-nanoparticles (Table 10).

### 2.16. Zinc and Iron Content

The zinc and iron contents were significantly affected by the independent effects of the treatments (Table 8). The salinity treatment reduced the Zn and Fe content compared to the control (Table 9). Higher Zn and Fe content (37% and 27%) was obtained in the plants supplemented with the foliar application of CeO_2_:SA-nanoparticles compared to the control (Table 10).

### 2.17. Na Content a Na/K Ratio

The results showed that the Na content and the Na/K ratio were significantly affected by the interactions of salinity stress and foliar applications (Table 8). The NaCl treatment enhanced the Na content and the Na/K ratio, but all foliar applications reduced these values. The lowest Na content was observed in the plants sprayed with CeO_2_:SA-nanoparticles. All the three foliar treatments reduced the Na uptake in the purslane plants under the salinity stress (Table 11).

### 2.18. Magnesium Content

The Mg content was affected by the salinity stress and foliar applications (Table 8). The 100 mM salinity decreased the Mg content, whereas the foliar application of salicylic acid and CeO_2_-nanoparticles increased the Mg amount in the plants, compared to the control. The highest Mg content was observed in the treatment with CeO_2_:SA-nanoparticles, which showed a 43% increase compared to the control (Table 11).

### 2.19. Manganese Content

The lowest Mn content was observed in the 100 mM salinity stress variant without foliar applications. All foliar treatments improved the Mn content at all levels of salinity stress. The highest Mn content was observed in the plants exposed to CeO_2_:SA-nanoparticles, in which the Mn content was enhanced by 96% compared to the control (Table 11).

### 2.20. Multivariate Analysis of NaCl Salinity × Foliar Use of Salicylic Acid, Cerium Oxide-NPs, and CeO_2_:SA-NPs Effects on Growth Responses in Portulaca Oleracea Plants

**Figure 1:**
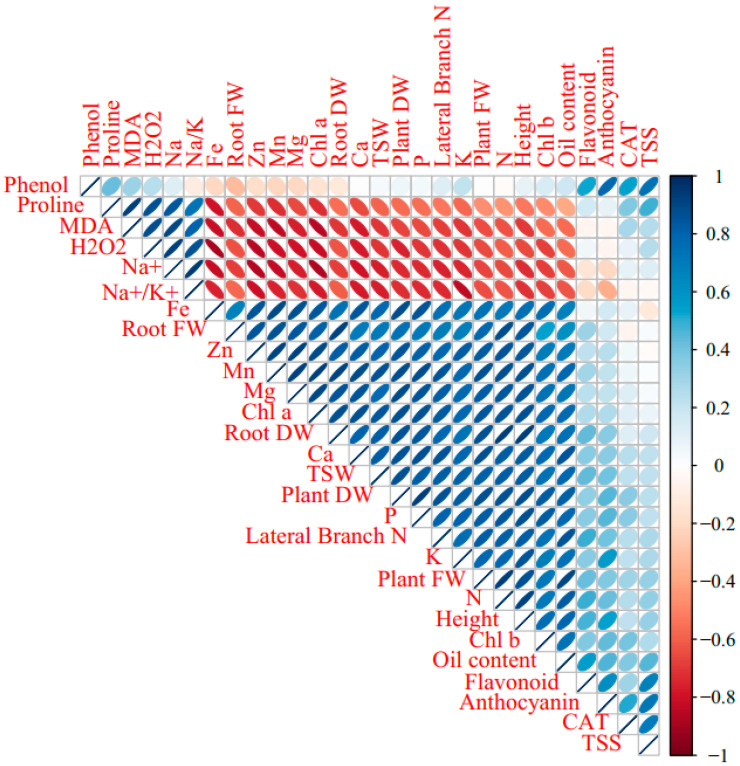
Pearson’s correlation heat map of NaCl salinity × foliar applications (distilled water, salicylic acid, cerium oxide nanoparticles, and CeO_2_:SA-nanoparticles) on the growth, physiological responses, and elemental content in *Portulaca oleracea* plants. FW & DW refers to fresh and dry weight, respectively.

The Pearson’s correlation heat map of the morphological and biochemical responses, nutrient elements, and antioxidant attributes is presented in Figure 1. The results indicated a significant positive correlation among Na, Na/K, H_2_O_2_, MDA, and proline. In turn, Na, Na/K, H_2_O_2_, MDA, and proline negatively correlated with Fe, Zn, Mn, Mg, Ca, P, K, and N content and with root fresh and dry weight, plant fresh and dry weight, branch number, plant height, oil yield, photosynthetic pigment content, and 1000-seed weight (TSW). The total soluble solid content was correlated with CAT activity and the content of anthocyanins, total phenolics, and flavonoids.

**Figure 2:**
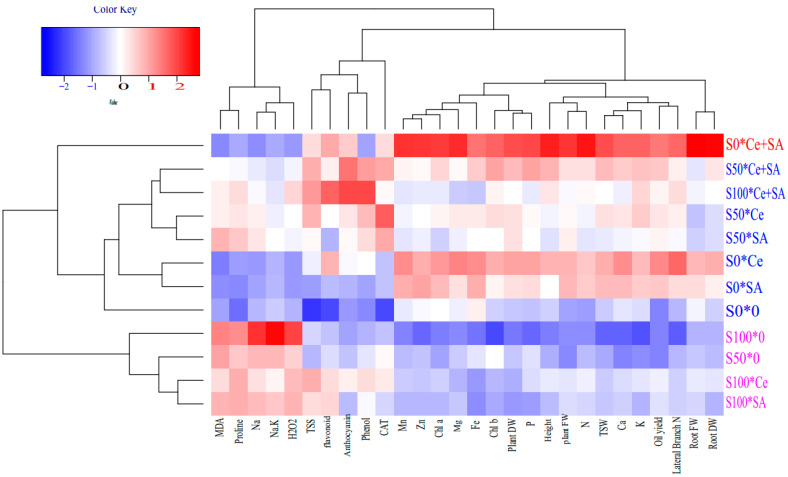
Heat map of the cluster analysis dendrogram for the effects of NaCl salinity and foliar applications (distilled water, salicylic acid, cerium oxide nanoparticles, and CeO_2_:SA-nanoparticles) on the growth, physiological responses, and elemental content in *Portulaca oleracea* plants. FW & DW refers to fresh and dry weight, respectively.

The heat maps (Figure 2) showed that the MDA, proline, Na, Na/K, and H_2_O_2_ had positive compliance with salinity. In turn, the salinity stress adversely affected such traits as the Fe, Zn, Mn, Mg, Ca, P, K, and N content, root fresh and dry weight, plant fresh and dry weight, branch number, plant height, oil yield, photosynthetic pigment content, and TSW. The heat maps revealed that salicylic acid and cerium oxide NPs modulated the harmful effect of salinity stress by improving some of the morphological and biochemical responses and content of nutritional elements.

The cluster analysis and dendrograms in the heat map revealed three main groups in the evaluated characteristics of *Portulaca oleracea* under salinity stress × foliar treatments. Group 1 contained the Fe, Zn, Mn, Mg, Ca, P, K, and N content, root fresh and dry weight, plant fresh and dry weight, branch number, plant height, oil yield, photosynthetic pigment content, and TSW. Group 2 contained the flavonoid content, phenolic content, CAT activity, and anthocyanin content. Group 3 contained proline, MDA, H_2_O_2_, Na, and Na/K. Groups 1 and 3 exhibited a negative correlation with each other. In general, the cluster analysis of the heat maps for the treatments showed three main groups. Group 1 contained the use of Ce + SA without salinity stress, and group 2 included Ce, SA, and Ce + SA under the 50 mM NaCl treatment, Ce and SA under the 0 mM NaCl treatment, the control, and, finally, Ce + SA with the 100 mM NaCl treatment. Group 3 included 50 and 100 mM NaCl and Ce + SA under 100 mM NaCl.

## 3. Discussion

The results showed the damaging effects of salinity stress on the growth and yield of *Portulaca oleracea* plants. He et al. [10] reported that the highest purslane yield (as a halophyte plant) was obtained under 100 mM salinity. In a study on two purslane genotypes, salinity adversely influenced the number of leaves due to salt accumulation in the aerial parts of plants [24]. It seems that the yield of purslane plants in a salinity environment is dependent on other growth stimuli, e.g., plant ecotype or cultivar, temperature, and light intensity [25]. In bean plants, the application of cerium oxide nanoparticles (250 mg L^−1^) increased plant yields due to the enhanced pollen grain genesis, ovary development, and enhanced grain protein content [26]. This was related to the reduction in oxidative damage to the ovaries and an improved photosynthesis rate [26,27,28]. Appropriate concentrations of cerium oxide intensify photosynthesis and plant growth by improving chlorophyll content [27,29]. Salicylic acid is a growth regulator essential in protecting the plant against biotic and abiotic stress factors. Foliar application of salicylic acid under salinity reduced SOD and catalase activity, improved potassium content, and increased rice yields [30]. In a study on sunflowers, salicylic acid under 200 mM salinity stress greatly improved plant growth. The increased plant growth induced by the application of salicylic acid is related to enhanced levels of antioxidants and metabolic activities, which increase resilience to stress [31].

The high specific surface area of nanoparticles and their small size facilitate their penetration into the cell and thus help to improve plant growth [32,33]. Considering the positive effect of CeO_2_ nanoparticles/SA on the fresh and dry weight and height of plants, it seems that their combined influence on several growth-related traits improves the yield and productivity of plants. Even though the most significant effect of the co-treatments was recorded in no-salinity conditions, the application of the nanoparticles under 50 and 100 mM salinity improved the plant yield compared to similar salinity treatments without foliar applications. The chlorophyll content may be considered a biochemical marker of salinity tolerance in plants; its variations reflect the plant’s responses to salinity conditions [24]. Numerous studies on different plants revealed that salinity stress alters the morphological and physiological responses by changing the sodium and potassium ion ratio, water relations, nutrient uptake, and stomatal closure, and hence considerably decreases the photosynthetic potential and yield of the plant [8]. More possibly, cerium oxide nanoparticles have functions in chlorophyll biosynthesis and the protection of chloroplast structure against salinity-induced defects [19,34,35]. A study on sweet pepper revealed that SA application (0.2 mM) under 60 mM salinity increased leaf area index, photosynthesis, chlorophyll content, plant dry weight, and SOD activity. SA activates the antioxidant defense mechanism, improves ion absorption, concomitantly reduces ethylene production inside plants, improves stomatal conductance, and helps the plants survive in stressful environments [36].

Salinity stress causes hyper-generation of oxygen free radicals, which have a destructive effect on the integrity of the cell membrane and the structure of phospholipids. Biological macromolecules face turbulence and sudden damage due to the excessive production of superoxide and hydroxyl radicals caused by salinity stress. Our results showed increased H_2_O_2_ and MDA contents under 100 mM salinity stress without foliar application. Similar results were reported by Kim et al. [37] in rice. Under salinity, the enhanced production of SA, ethylene, and jasmonic acid triggers signaling cascades as defense mechanisms versus stress factors that control several molecular, biochemical, and physiological pathways in plants, mitigates the effects of the stressors, and gives plants high endurance in extremely stressful environments [37]. Similar to our results, in *Brassica napus* L., CeO_2_ nanoparticles treatment reduced the MDA and H_2_O_2_ content in the plants [38].

Plants are equipped with enzymatic and non-enzymatic defense systems to maintain the homeostasis of free radicals. The pioneer enzymatic defense system includes the activity of SOD and CAT enzymes. SOD is in the front line of defense against free radicals and manages the elimination of superoxide radicals [39]. The SA effect in regulating the antioxidant defense system depends on the plant genotype and the time of SA consumption [40]. The results of studies on *Brassica carinata* [41], *Dianthus superbus* [42], and *Sorghum bicolor* [43] showed that SA increased catalase activity in the plants, which indicates the positive role of SA in modulating cellular redox balance and protecting the plant against oxidative damage.

Proline, phenolics, and soluble solids play a predominant action in scavenging oxygen free radicals and protection against stress factors [44]. Treatment of plants with cerium oxide nanoparticles in flax [18] and rapeseed [32] improved plant growth, increased the content of phenolic compounds, chlorophyll, and plant yield, and reduced malondialdehyde and H_2_O_2_ content. Similar results, i.e., an increase in the content of phenolic compounds due to salinity stress, have been reported in safflower [31] and *P. oleraceae* [10]. A study in lettuce showed that cerium oxide nanoparticles increased the content of gallic acid and vanillic acid, clearly representing the positive effect of cerium oxide on the biosynthesis and accumulation of phenolic compounds [44]. Application of salicylic acid under 100 mM salinity stress increased plant yield, phenolic content, chlorophyll biosynthesis, and antioxidant properties of *Salvia coccineae* [45].

Furthermore, salicylic acid (100 μM) under salinity stress did not affect the anthocyanin content in strawberries [34]. Still, the stress increased the content of total phenolics and soluble solids in the plant. It seems that the increase in the content and activity of compatible solutes in response to foliar application of salicylic acid establishes metabolic balance, modulates free radical levels in the plant, maintains photosynthetic capacity, prevents membrane degradation, and maintains chloroplast structure in stress conditions, which are crucially important in protection in stressful environments [20,35,46,47].

Significant correlations were determined between salinity and proline content. Salinity stimulates the expression of genes related to proline biosynthesis [47,48,49]. In the current study, salinity enhanced the proline content, which was in agreement with results reported by Sdouga et al. [46]. In stressful environments, proline functions as an energy source, a compatible osmolyte, and an antioxidant to protect the cells and tissues against stressors’ side effects. In stress conditions, proline accumulation contributes to osmotic adjustment in the cell and protects membrane integrity [42].

Purslane plants are rich in nutrients, e.g., K, Ca, Mg, and Fe [10]. The foliar treatments used in the present study reduced the ratio of sodium to potassium, which clearly showed their effectiveness in reducing the sodium content in the plants. The salinity stress reduced the K, Ca, Fe, and Zn content in the plants. In another study, high salinity levels decreased the content of K, Ca, and Mg in purslane. However, at high salinity levels (300 mM), the Fe content of plants was increased [10]. A possible cause of the reduced mineral uptake in the salinity conditions may be the particular root system architecture of purslane plants which produce small roots with low expansion rates [10]. Maintenance of a balanced Na and K homeostasis is critical for cell survival in stress conditions. Potassium activates various enzymes and regulates cytosolic pH, protein biosynthesis, cellular activity, and stomatal behavior. In stress conditions, plants lose more than 50% of their potassium [8,17,44]. Cerium oxide nanoparticle treatment under salinity increased the potassium content and decreased the cytosolic sodium content. Furthermore, a decrease in the activity of root apoplastic carriers mediating sodium ion absorption in response to cerium oxide treatment has been reported to enhance plant tolerance to stress factors [50]. However, the treatment of plants with salicylic acid improved plant growth, fresh and dry weight, TSS content, and potassium and calcium content [22,43]. There is a positive correlation between internal salicylic acid levels and tolerance to salinity stress in halophyte plants. In stress conditions, salicylic acid acts as a signaling molecule and plays an essential role in activating plant defense mechanisms [22,25].

Nanoparticles mitigate the effect of free radicals and have a crucial function in establishing free radical homeostasis and improving plant responses under stress [17]. Treatment with cerium oxide nanoparticles modulates the activity of ROS-activated NSCC channels (non-selective cation channels) and KOR (K^+^ outward rectifying channel) to maintain sufficient amounts of K^+^ in plants under salinity stress [17,47,48]. In stress conditions, an increased K/Na ratio improves plant resistance to stress. In flaxseed plants growing under salinity, cerium oxide nanoparticles treatment caused upstream regulation of the HKT1 gene, which played an important role in excluding sodium ions from the cell [17]. Some studies have shown that, following the elimination of free radicals by cerium oxide nanoparticles in stress conditions, this substance can regulate the activity of protein channels associated with potassium efflux, thereby helping to maintain sufficient amounts of potassium in the cell [47,48]. The foliar application of cerium oxide nanoparticles under salinity stress in *Brassica napus* increased potassium content by 29% and the K/Na ratio by 37%, compared to control plants [32]. Under stress, appropriate nutrient uptake via regulating photosynthetic processes, the antioxidant defense system, and enzymatic activity improves cell and plant function, contributing to efficient survival in stress conditions [8,24,49].

In general, nearly all the growth-related traits such as plant fresh and dry weight, root fresh and dry weight, number of branches, physiological attributes, antioxidant enzyme activities, and mineral content were influenced by the salinity stress. The mitigating function of the foliar applications and especially the ameliorative effects of the combined foliar treatment were promising in compensating salinity defects. The positive roles of the foliar treatments may be ascribed to the tiny size of the nanoparticles, facilitating their absorption, translocation, function, and metabolism. Indeed, nanoparticles have frequently evidenced their efficacy in stressful environments. SA is also a predominant signaling molecule whose stress-induced growth-promoting functions are clearly evident. Therefore, the combined application of SA and cerium oxide nanoparticles will intensify their positive effect. The results from the current study indicate the relevance of the joint application of these compounds and maybe their counterparts to combat the stress impact effectively and to ensure the production of valuable plants in harsh environments.

## 4. Materials and Methods

This experiment was conducted during the spring and summer of 2020 at the Research Greenhouse of Azarbaijan Shahid Madani University, Tabriz, Iran. The greenhouse environmental conditions were as follows: lightening period: 16:8 day and night, temperature regime: 30:25 day and night, and approx. 70% relative humidity.

### 4.1. Plant Material and Experimental Setup

*Portulaca oleracea* seeds were surface sterilized with sodium hypochlorite (10%) for 10 min, followed by washing with distilled water 3 times. The seeds (local clone supplied by Pakan Bazr Seed Company, Esfahan, Iran) were planted in 5-L pots containing medium-sized perlite. During the early growth stages and acclimation, the plants were nourished with half-strength Hoagland’s nutrient solution. When the plants had three real leaves, salinity treatment was imposed. The salinity levels were 0, 50, and 100 mM of NaCl. The lowest salinity level was 25 mM, which gradually increased to reach the final level within ten days [9]. The optimal pH of the nutrient solution (NS) was 5.8 and was recorded every day and adjusted accordingly using H_2_SO_4_ (5% *v*/*v*). Following the salinity application, the EC of the nutrient solution was 2.1 mS cm^−1^ (0 mM NaCl), 6.0 mS cm^−1^ (50 mM NaCl), and 12.0 mS cm^−1^ (100 mM NaCl). The pots were regularly washed with tap water once every week to avoid salt deposition on the pot surfaces. Foliar treatments were applied as sprays, and four solutions were used: dH_2_O, 100 µM salicylic acid, 50 mg L^−1^ cerium oxide nanoparticles, and cerium oxide: salicylic acid nanoparticles (CeO_2_:SA-NPs; 100 µM + 50 mg L^−1^) [50]. Foliar applications were applied twice. The first application was performed just after salinity exposure (3rd-leaf stage), and the second foliar spray was applied two weeks later. The plant samples were further analyzed two months after the second foliar treatment. The experiment consisted of 12 treatment combinations and 3 replications. Each experimental unit had 4 pots. Each pot contained 4 plants. In total, the experiment was composed of 144 pots with 576 plants. The pots were spaced 50 cm between rows and 20 cm within rows. The pots were manually irrigated with 250 mL of the nutrient solution every 5 days.

### 4.2. Synthesis of CeO_2_ Nanoparticles

The CeO_2_ nanoparticles were synthesized via a facile sonochemical procedure. The precursor materials used in this research were Ce(NO_3_)_3_ hexahydrate and urea. A total of 0.05 M of (Ce(NO_3_)_3_,6H_2_O) was dissolved in 17 mL of water and ultra-sonicated for 20 min. After that, 20 g urea was added to the solution and allowed to sonicate for 2 h. Subsequently, the solution was centrifuged (8000 rpm/15 min) and washed with water and ethanol to remove the unreacted materials. Finally, the precipitate material was dried at 50 °C overnight. Later, the dried powder was calcined at 850 °C for 5 h.

### 4.3. Preparation of CeO_2_:SA-Nanoparticles

CeO_2_-NPs were mixed with salicylic acid by molar ratios (1:0 1:1, 1:2, 2:1, and 0:1) with 0, 50, and 100 µm. For example, the 500 mL solution of CeO_2_ nanoparticles (50 µm) and salicylic acid (50 µm) (1:1) was fabricated by a sonication bath at 60 °C for 1 hour and then continued by pulse probe sonication for 1 more hour to form a good clear solution without any suspended particles.

### 4.4. Transmission Electron Microscopy (TEM) and Dynamic Light Scattering (DLS) Analysis

**Figure 3:**
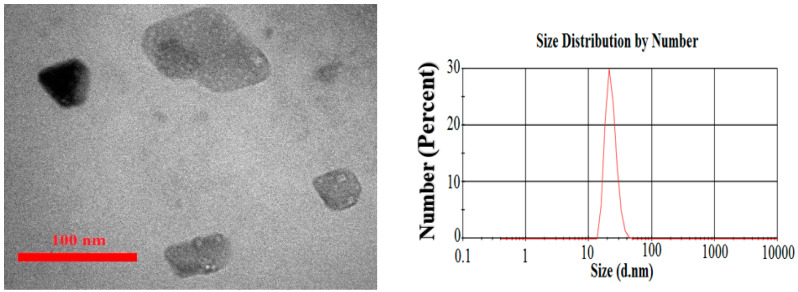
TEM image of sonochemical synthesis of CeO_2_-SA nanoparticles (**left**), DLS analysis of CeO_2_-SA nanoparticles (**right**).

Figure 3 (left) represents the TEM image of fabricated CeO_2_: SA-NPs. The successful octahedron nanoparticles are around 30–80 nm, which agrees with the DLS data presented in Figure 3 (right).

### 4.5. Fresh and Dry Weight of Plants

The fresh and dry biomass of plant organs was recorded using a digital scale (BB141, Boeco, Germany). After harvesting, the above- and underground plant parts were separated and air-dried until reaching constant weight.

### 4.6. Chlorophyll Content

Chlorophylls a and b were quantified as in Prochazkova et al. [51] at 645 and 665 nm. Leaf samples (0.5 g for each replication) were extracted using dimethyl sulfoxide (DMSO, Sigma Aldrich, Germany) in the dark for 4 h at 65 °C

### 4.7. Total Soluble Solid Content (°Brix)

The content of Total Soluble Solids (TSS) in the leaves (1 g for each replication) was quantified with a hand refractometer (Erma, Tokyo, Japan), and the data are presented as °Brix.

### 4.8. Oil Extraction

The fatty oil yield in seeds was quantified using a diethyl-ether (DEE) solution. A total of 1 gram of ground, dried (60 °C for 24 h) seed from each replication was poured into glass vials. Then, 10 mL of DEE was added and vortexed to acquire a homogenous solution. The solution was transferred to 50 mL falcons and centrifuged for 10 min at 10,000 rpm. The supernatant, i.e., diethyl ether and dissolved fatty oils, were collected and transferred to another pre-weighed vial. The vials were placed in an oven at 30 °C for 24 h to remove the solvent. The remaining was pure oil, and the oil yield was calculated correspondingly.

### 4.9. Content of Phenolics and Flavonoids

A methanolic extract of plant tissue (0.5 g dry methanolic extract) was used to quantify the phenolic content by Folin–Ciocalteu reagent at 755 nm, according to Kim et al. [52]. The results were expressed as equivalents of gallic acid (Scharlau, Barcelona, Spain) per g of plant dry weight (mg of GAE/g dry weight).

Total flavonoids were assayed with the aluminum chloride colorimetric method at 510 nm, as described by Kim et al. [52]. The results of total phenolic compounds were expressed as equivalent of gallic acid (Scharlau, Barcelona, Spain) per g of plant dry weight (mg of GAE/g dry weight), and total flavonoids were expressed as rutin equivalents (mg Rutin g^−1^ dry weight).

### 4.10. Anthocyanin Content

Total anthocyanins were determined in fresh leaf (0.5 g for each replication) tissues. The leaves were homogenized in acidified (HCl) methanol. A spectrophotometer measured the absorption of anthocyanins at 550 nm, as in Wanger [53].

### 4.11. Proline Content

A total of 5 mL of 3% homogenized sulfosalicylic acid was added to 0.2 g of leaf sample. The extract was centrifuged at 6000 rpm for 7 min. Then, 1 mL of the supernatant was mixed with the same volume of ninhydrin acid and 1 mL of glacial acetic acid. Later, the samples were incubated in a 100 °C water bath and then in an ice bath for 30 s. After 30 min, the red phase formed above the sample was used for the proline content measurements at 520 nm, based on the method proposed by Fedina et al. [54]. The proline content was computed using a standard curve of proline, and the results were expressed as micromol of proline per gram of fresh plant weight.

### 4.12. Hydrogen Peroxide Content and Lipid Peroxidation

The content of H_2_O_2_ was assessed according to Alexieva et al. [55]. A total of 0.2 g of leaf tissue was powdered in liquid N_2_ and then ground in ice-cold 0.1% trichloroacetic acid (TCA) and centrifuged at 12,000× *g* for 15 min. An aliquot (0.5 mL) of the supernatant was mixed with 0.5 mL of 10 mM potassium phosphate buffer (pH = 7.5) and 1 mL of 1M potassium iodide. The H_2_O_2_ content was evaluated using standards of 5 to 1000 μM of H_2_O_2_, and the calibration curve was plotted accordingly. The absorbance of samples and standards was measured at 390 nm, and results were expressed as μmol H_2_O_2_ g^−1^ fresh weight.

Lipid peroxidation was determined regarding the malondialdehyde (MDA) content, as described by Sahu and Sabat [56]. Leaf tissue (0.2 g) was homogenized in 0.1% TCA, and the extract was centrifuged at 12,000× *g* for 15 min. The reaction mixture of 0.5 mL of the extract and 1.5 mL of 0.5% thiobarbituric acid (TBA) in 20% TCA was incubated at 95 °C for 30 min and then cooled in an ice bath. The absorbance was determined at 520 nm and corrected for non-specific absorbance at 600 nm. The MDA amount was determined using the extinction coefficient of 155 mM cm^−1^. The results were expressed as nmol of MDA g^−1^ fresh weight.

### 4.13. Catalase Activity

A total of 0.5 g of purslane leaf samples were homogenized with 0.1 M cold potassium phosphate buffer (pH: 7.5) with 0.5 mM EDTA based on the method developed by Luhova et al. [57]. From the resulting supernatant, 0.05 mL was added to 1.5 mL of 0.1 mM phosphate buffer (pH: 7) and 1.45 mL of double-distilled water. The reaction was started by adding 0.5 mL of 75 mM hydrogen peroxide, and a decrease in absorption was recorded at 240 nm for 1 min [57].

### 4.14. Elemental Composition

The leaves (3 replications/treatment) were dried at 75 °C for a day, weighted, and ground in a Wiley mill to particles less than 0.42 mm. Subsamples (0.2–0.3 g) were acid digested (2 N HCl) and analyzed for nutrient content as described by Chrysargyris et al. [9]. The content of Na and K was quantified with the flame photometric method (Corning, 410, England). The amounts of Mn, Mg, Zn, Ca, and Fe were measured by atomic absorption spectroscopy (Shimadzu, AA6300, Tokyo, Japan) as previously described by Honarjoo et al. (2013). Phosphorus was determined with the Vanadate Molybdate method [58], and the N content was assessed with the Kjeldahl method [58].

### 4.15. Experimental Design and Data Analysis

The experiment was conducted as a factorial based on RCBD with three replications. Analysis of variance (ANOVA) was performed by MSTAT-C ver 2.1. Moreover, the significant differences among the means were evaluated with the Least Significance Difference test (LSD) at *p* < 0.05 and *p* < 0.01. Standard deviations (n = 3) were evaluated for the traits. Pearson’s correlation and cluster dendrogram heat maps were depicted in R software (R foundation for statistical computing, version 4.1.2).

## 5. Conclusions

Salinity stress reduced the yield, chlorophyll content, 1000-seed weight, catalase activity, and elemental content in *Portulaca oleracea*. The highest fresh and dry weight of plants was recorded in the no-salinity × CeO_2_:SA-nanoparticles foliar application variant. The maximum number of sub-branches was observed in the foliar treatment with CeO_2_-nanoparticles and CeO_2_:SA-nanoparticles without salinity stress. The TSS content, oil yield, pigment content, antioxidant enzyme activity, and elemental content were responsive to the salinity and foliar applications. CeO_2_:SA-nanoparticles effectively mitigated the adverse salinity effect and partially enhanced the growth and physiological attributes of purslane plants.

## Figures and Tables

**Table 1:** ANOVA for the effect of salinity (0–50 and 100 mM NaCl) and foliar applications (no spray, salicylic acid, CeO_2_-nanoparticles, and CeO_2_:SA-nanoparticles) on *Portulaca oleracea* growth characteristics, oil yield, and content of total soluble solids.

	Plant Fresh Weight (Fresh Biomass)	Root Fresh Weight	Plant Dry Weight (Dry Biomass)	Root Dry Weight	Plant Height	Branch Number	1000-Seed Weight	Total Soluble Solid Content	Oil Yield
**Salinity (S)**	**	**	**	**	**	**	**	**	**
**Foliar (F)**	**	**	**	**	**	**	**	**	**
**S × F**	**	**	**	**	**	**	ns	ns	ns

Significant effects for the main factors and their interaction are indicated with an asterisk: ** *p* < 0.01; ns: non-significant.

**Table 2:** Comparison of means for the effects of salinity (0–50 and 100 mM NaCl) and foliar applications (no spray, salicylic acid, CeO_2_-nanoparticles, and CeO_2_:SA-nanoparticles) on the growth characteristics of *Portulaca oleracea* plants.

Salinity	Foliar Spray	Plant Dry Weight (g)	Plant Fresh Weight (g)	Root Fresh Weight (g)	Root Dry Weight (g)	Plant Height (cm)	Sub-Branch Number
**0**	**No spray**	1.8 ± 0.07f	15.2 ± 1.28f	0.24 ± 0.02c	0.020 ± 0.0e–g	21.8 ± 1.02ef	4.0 ± 0.81d
**0**	**Salicylic acid**	2.8 ± 0.09d	27.5 ± 1.50b	0.38 ± 0.07b	0.065 ± 0.0c	25.0 ± 1.41cd	5.6 ± 0.47b
**0**	**CeO_2_-nanoparticles**	3.5 ± 0.09b	27.9 ± 2.56b	0.46 ± 0.05b	0.110 ± 0.0b	30.3 ± 1.25b	7.3 ± 0.47a
**0**	**CeO_2_: SA-nanoparticles**	4.5 ± 0.13a	36.9 ± 2.26a	0.91 ± 0.11a	0.180 ± 0.0a	39.6 ± 1.70a	7.3 ± 0.47a
**50**	**No spray**	1.8 ± 0.25f	13.2 ± 0.52f	0.13 ± 0.02d–f	0.180f ± 0.0g	19.4 ± 1.18g	4.0 ± 0.82d
**50**	**Salicylic acid**	2.8 ± 0.12d	23.6 ± 0.60cd	0.16 ± 0.01c–f	0.034 ± 0.0ef	23.0 ± 1.00de	5.0 ± 0.47bc
**50**	**CeO_2_-nanoparticles**	2.8 ± 0.12d	22.8 ± 1.51c–e	0.12 ± 0.01ef	0.035 ± 0.0ef	24.3 ± 0.05d	5.3 ± 0.47b
**50**	**CeO_2_: SA-nanoparticles**	3.2 ± 0.05c	24.6 ± 3.26bc	0.21 ± 0.06c–e	0.070 ± 0.0c	30.3 ± 0.94b	5.3 ± 0.82b
**100**	**No spray**	0.9 ± 0.04h	13.5 ± 1.76f	0.09 ± 0.01f	0.012 ± 0.0g	16.3 ± 0.94h	2.6 ± 0.82e
**100**	**Salicylic acid**	1.3 ± 0.08g	19.4 ± 0.58e	0.17 ± 0.02c–f	0.013 ± 0.0g	20.3 ± 0.93fg	4.3 ± 0.47cd
**100**	**CeO_2_-nanoparticles**	1.5 ± 0.14g	19.8 ± 0.87de	0.22 ± 0.03cd	0.041d ± 0.0e	23.5 ± 0.48de	4.3 ± 0.47cd
**100**	**CeO_2_: SA-nanoparticles**	2.4 ± 0.12e	21.4 ± 2.51c–e	0.25 ± 0.02c	0.050 ± 0.0cd	27.0 ± 0.82c	5.6 ± 0.47b

Means ± SD (n = 3) with different letters within the same column are statistically different according to the LSD test (*p* < 0.05).

**Table 3:** Comparison of means for the effect of NaCl salinity on 1000-seed weight, total soluble solid content, chlorophyll content, and oil yield of Portulaca oleracea plants.

Salinity	1000-Seed Weight	Total Soluble Solid Content (°Brix)	Oil Yield (g m^−2^)	Chlorophyll a Content (mg g^−1^FW)	Chlorophyll b Content (mg g^−1^FW)
**0**	3.0 ± 0.08a	1.6 ± 0.12b	0.64 ± 0.05a	2.40 ± 0.14a	0.95 ± 0.12a
**50**	2.7 ± 0.31ab	2.1 ± 0.08a	0.52 ± 0.09b	1.66 ± 0.12b	0.89 ± 0.17ab
**100**	2.4 ± 0.26b	2.2 ± 0.09a	0.45 ± 0.04b	1.33 ± 0.31b	0.65 ± 0.18b

Means ± SD (n = 3) with different letters within the same column are statistically different according to the LSD test (*p* < 0.05).

**Table 4:** Comparison of means for the effect of foliar applications on 1000-seed weight, total soluble solid content, chlorophyll content, and oil yield of Portulaca oleracea plants.

Foliar Application	1000-Seed Weight (g)	Total Soluble Solid Content (°Brix)	Oil Yield (g m^−2^)	Chlorophyll a Content (mg g^−1^FW)	Chlorophyll b Content (mg g^−1^FW)
**No spray**	2.2 ± 0.20c	1.5 ± 0.05c	0.26 ± 0.08b	1.28 ± 0.17b	0.66 ± 0.09c
**Salicylic acid**	2.7 ± 0.05b	1.9 ± 0.08b	0.56 ± 0.04a	1.68 ± 0.33b	0.78 ± 0.07b
**CeO_2_-nanoparticles**	2.8 ± 0.09ab	2.2 ± 0.03a	0.63 ± 0.09a	1.96 ± 0.21ab	0.84 ± 0.17b
**CeO_2_: SA-nanoparticles**	2.3 ± 0.08a	2.3 ± 0.09a	0.68 ± 0.06a	2.30 ± 0.33a	1.04 ± 0.09a

Means ± SD (n = 3) with different letters within the same column are statistically different according to the LSD test (*p* < 0.05).

**Table 5:** ANOVA for the effect of salinity (0–50 and 100 mM NaCl) and foliar applications (no spray, salicylic acid, CeO_2_-nanoparticles, and CeO_2_: SA-nanoparticles) on the content of chlorophylls, total phenolics, flavonoids, and anthocyanins in *Portulaca oleracea* plants.

	Chlorophyll a Content	Chlorophyll b Content	Total Phenolic Content	Flavonoid Content	Anthocyanin Content
**Salinity (S)**	**	**	**	**	**
**Foliar (F)**	**	**	**	**	**
**S × F**	ns	ns	**	**	*

Significant effects for the main factors and their interaction are indicated with an asterisk: * *p* < 0.05; ** *p* < 0.01; ns: non-significant.

**Table 6:** Comparison of means for the effects of salinity (0–50 and 100 mM NaCl) and foliar applications (no spray, salicylic acid, CeO_2_-nanoparticles, and CeO_2_:SA-nanoparticles) on some biochemical characteristics of *Portulaca oleracea* plants.

Salinity	Foliar Spray	Phenolic Content (mg g^−1^ DW)	Flavonoid Content (mg g^−1^ DW)	Anthocyanin Content (mg g^−1^ FW)	MDA Content (µmol g^−1^ FW)	H_2_O_2_ Content (µmol g^−1^ FW)	CAT Activity (nmol H_2_O_2_ mg Protein m^−1^)	Proline Content (µmol g^−1^ FW)
**0**	**No spray**	38.67 ± 0.47f	4.00 ± 0.81g	1.56 ± 0.36d	20.67 ± 2.05f	1.33 ± 0.16e	0.30 ± 0.08f	1.93 ± 0.23g
	**Salicylic acid**	43.00 ± 1.63ef	6.33 ± 0.47ef	1.96 ± 0.12d	17.33 ± 1.69ef	1.07 ± 0.12e	0.53 ± 0.04e	2.67 ± 0.24fg
	**CeO_2_-nanoparticles**	58.67 ± 2.05d	9.50 ± 1.08ac	3.16 ± 0.44c	14.67 ± 0.77f	1.06 ± 0.09e	0.53 ± 0.06e	3.23 ± 0.47f
	**CeO_2_: SA-nanoparticles**	43.67 ± 1.24ef	9.66 ± 0.99ab	4.23 ± 0.20b	16.33 ± 2.49f	1.07 ± 0.16e	0.73 ± 0.07bc	3.47 ± 0.38f
**50**	**No spray**	54.67 ± 3.68d	7.00 ± 0.41df	2.20 ± 0.28d	52.00 ± 2.94b	2.63 ± 0.28bc	0.66 ± 0.04ce	7.60 ± 0.29bc
	**Salicylic acid**	66.33 ± 1.24c	6.16 ± 0.23f	3.43 ± 0.30bc	49.67 ± 3.29b	2.00 ± 0.14d	0.83 ± 0.09ab	7.57 ± 0.55bd
	**CeO_2_-nanoparticles**	71.00 ± 3.55c	7.90 ± 0.53ce	3.83 ± 0.12bc	39.33 ± 1.24cd	2.16 ± 0.23d	0.96 ± 0.08a	6.67 ± 0.49de
	**CeO_2_: SA-nanoparticles**	76.67 ± 4.66b	8.20 ± 0.80bd	5.70 ± 0.49a	35.67 ± 0.94d	2.03 ± 0.18d	0.83 ± 0.03ab	5.77 ± 0.25e
**100**	**No spray**	45.67 ± 3.29e	6.50 ± 0.40ef	1.70 ± 0.43b	57.33 ± 2.05a	4.03 ± 0.27a	0.53 ± 0.04e	9.13 ± 0.12a
	**Salicylic acid**	58.33 ± 2.05d	8.66 ± 0.47bd	2.13 ± 0.12d	49.33 ± 1.24b	2.97 ± 0.09b	0.56 ± 0.11de	8.30 ± 0.24ab
	**CeO_2_-nanoparticles**	66.33 ± 3.09c	8.56 ± 0.75bd	3.67 ± 0.09bc	43.00 ± 1.69c	2.87 ± 0.12bc	0.70 ± 0.10bd	8.20 ± 0.49b
	**CeO_2_: SA-nanoparticles**	91.00 ± 1.63a	11.10 ± 0.69a	6.43 ± 0.71a	39.33 ± 1.24cd	2.57 ± 0.10c	0.66 ± 0.70cd	7.00 ± 0.81cd

Means ± SD (n = 3) with different letters within the same column are statistically different according to the LSD test (*p* < 0.05).

**Table 7:** ANOVA for the effect of salinity (0–50 and 100 mM NaCl) and foliar applications (no spray, salicylic acid, CeO_2_-nanoparticles, and CeO_2_:SA-nanoparticles) on the MDA, proline, and H_2_O_2_ content and CAT activity in *Portulaca oleracea* plants.

	Malondialdehyde Content	H_2_O_2_ Content	Catalase Activity	Proline Content
**Salinity (S)**	**	**	**	**
**Foliar (F)**	**	**	**	**
**S×F**	**	**	*	**

Significant effects for the main factors and their interaction are indicated with an asterisk: * *p* < 0.05; ** *p* < 0.01; ns: non-significant.

**Table 8:** ANOVA for the effect of salinity (0–50 and 100 mM NaCl) and foliar applications (no spray, salicylic acid, CeO_2_-nanoparticles, and CeO_2_: SA-nanoparticles) on the elemental content in *Portulaca oleracea* plants.

	N Content	P Content	K Content	Na Content	K/Na Ratio	Ca Content	Mg Content	Fe Content	Zn Content	Mn Content
**Salinity (S)**	**	**	**	**	**	**	**	**	**	**
**Foliar (F)**	**	**	**	**	**	**	**	**	**	**
**S × F**	**	*	ns	**	**	ns	**	ns	ns	**

Significant effects for the main factors and their interaction are indicated with an asterisk: * *p* < 0.05; ** *p* < 0.01; ns: non-significant.

**Table 9:** Comparison of means for the effects of salinity on K, Ca, Fe, and Zn content in *Portulaca oleracea* plants.

Salinity	K Content (g kg^−1^ DW)	Ca Content (g kg^−1^ DW)	Fe Content (mg kg^−1^ DW)	Zn Content (mg kg^−1^ DW)
**0**	19.50 ± 1.25a	22.80 ± 1.22a	244 ± 8.10a	73 ± 4.11a
**50**	17.10 ± 1.88ab	17.50 ± 0.80b	204 ± 14.90b	58 ± 3.41b
**100**	14.80 ± 2.32b	14.90 ± 0.92c	145 ± 19.00c	50 ± 2.14c

Means ± SD (n = 3) with different letters within the same column are statistically different according to LSD test (*p* < 0.05).

**Table 10:** Comparison of means for the effects of foliar applications (no spray, salicylic acid, CeO_2_-nanoparticles, and CeO_2_:SA-nanoparticles) on K, Ca, Fe, and Zn content in *Portulaca oleracea* plants.

Foliar Spray	K Content (g kg^−1^ DW)	Ca Content (g kg^−1^ DW)	Fe Content (mg kg^−1^ DW)	Zn Content (mg kg^−1^ DW)
**No spray**	12.80 ± 2.46c	13.00 ± 1.63c	173 ± 7.41c	50.3 ± 2.40c
**Salicylic acid**	16.70 ± 1.41b	18.30 ± 1.48b	193 ± 6.49b	60.0 ± 3.40b
**CeO_2_-nanoparticles**	18.50 ± 0.81ab	20.00 ± 0.90ab	204 ± 12.39ab	61.0 ± 1.25ab
**CeO_2_:SA-nanoparticles**	20.60 ± 0.94a	21.30 ± 0.85a	221 ± 9.34a	69.0 ± 7.91a

Means ± SD (n = 3) with different letters within the same column are statistically different according to the LSD test (*p* < 0.05).

**Table 11:** Comparison of means for the effects of salinity (0–50 and 100 mM NaCl) and foliar applications (no spray, salicylic acid, CeO_2_-nanoparticles, and CeO_2_: SA-nanoparticles) on the content of some nutrients in *Portulaca oleracea* plants.

Salinity	Foliar Spray	N (g Kg^−1^ DW)	P (g Kg^−1^ DW)	Na (mg Kg^−1^ DW)	Na/k	Mg (mg Kg^−1^ DW)	Mn (mg Kg^−1^ DW)
**0**	**No spray**	6.67 ± 0.47gh	0.73 ± 0.12ef	2.17 ± 0.30g	0.14 ± 0.01fg	20.67 ± 0.47de	17.67 ± 2.05de
	**Salicylic acid**	14.73 ± 1.59b	1.07 ± 0.09bc	1.73 ± 0.09gh	0.09 ± 0.00gh	24.33 ± 2.86c	25.67 ± 2.49b
	**CeO_2_-nanoparticles**	14.97 ± 1.25b	1.23 ± 0.04b	1.57 ± 0.09gh	0.078 ± 0.00gh	31.00 ± 1.63b	28.00 ± 0.81b
	**CeO_2_: SA-nanoparticles**	23.70 ± 0.95a	1.50 ± 0.08a	1.27 ± 0.28h	0.05 ± 0.00h	36.67 ± 1.69a	34.67 ± 0.94a
**50**	**No spray**	8.27 ± 0.44fg	0.83 ± 0.10df	5.53 ± 0.40b	0.43 ± 0.03d	18.33 ± 0.94ef	14.33 ± 2.47f
	**Salicylic acid**	10.37 ± 0.62e	0.93 ± 0.09ce	4.53 ± 0.54de	0.26 ± 0.02de	20.90 ± 1.04de	17.33 ± 0.94de
	**CeO_2_-nanoparticles**	11.17 ± 0.62de	0.96 ± 0.11cd	4.30 ± 0.63de	0.22 ± 0.01ef	23.73 ± 0.61c	18.33 ± 0.67cd
	**CeO_2_: SA-nanoparticles**	13.67 ± 0.85bc	1.23 ± 0.04b	3.37 ± 0.59f	0.17 ± 0.02f	22.73 ± 1.25cd	20.33 ± 0.92c
**100**	**No spray**	6.03 ± 0.49h	0.46 ± 0.06g	8.37 ± 0.41a	0.81 ± 0.08a	13.33 ± 1.24g	10.00 ± 1.63g
	**Salicylic acid**	9.60 ± 0.57ef	0.63 ± 0.07fg	5.40 ± 0.49bc	0.39 ± 0.08cd	17.67 ± 0.47f	13.67 ± 0.94f
	**CeO_2_-nanoparticles**	10.97 ± 0.84de	0.81 ± 0.02df	4.57 ± 0.24cd	0.28 ± 0.04d	16.50 ± 1.22f	15.13 ± 0.94ef
	**CeO_2_: SA-nanoparticles**	12.30 ± 1.12cd	0.86 ± 0.05ce	3.70 ± 0.43ef	0.19 ± 0.01ef	18.27 ± 0.95ef	17.27 ± 1.26de

Means ± SD (n = 3) with different letters within the same column are statistically different according to the LSD test (*p* < 0.05).

## Data Availability

All-new research data were presented in this contribution.

## References

[1] Zhou Y.X., Xin H.L., Rahman K., Wang S.J., Peng C., Zhang H. (2015). *Portulaca oleracea* L.: A review of phytochemistry and pharmacological effects. Biomed Res. Int..

[2] Dabbou S., Lahbib K., Pandino G., Dabbou S., Lombardo S. (2020). Evaluation of pigments, phenolic and volatile compounds, and antioxidant activity of a spontaneous population of *Portulaca oleracea* L. grown in Tunisia. Agriculture.

[3] https://www.fao.org/newsroom/detail/salt-affected-soils-map-symposium/en#:~:text=Rome%20%2D%20The%20Food%20and%20Agriculture,8.7%25%20of%20the%20planet.

[4] Vojodi Mehrabani L., Valizadeh Kamran R., Hassanpouraghdam M.B., Pessarakli M. (2017). Zinc sulfate foliar application effects on some physiological characteristics and phenolic and essential oil contents of *Lavandula stoechas* L. under sodium chloride (NaCl) salinity conditions. Commun. Soil Sci. Plant Anal..

[5] Acosta-Motos J.R., Ortuño M.F., Bernal-Vicente A., Diaz-Vivancos P., Sanchez-Blanco M.J., Hernandez J.A. (2017). Plant responses to salt stress: Adaptive mechanisms. Agronomy.

[6] Akhavan Hezaveh T., Pourakbar L., Rahmani F., Alipour H. (2019). Interactive effects of salinity and ZnO nanoparticles on physiological and molecular parameters of rapeseed (*Brassica napus* L.). Commun. Soil Sci. Plant Anal..

[7] Faizan M., Bhat J.A., Chen C., Aleymeni M.N., Wijaya L., Ahmad P., Yu F. (2021). Zinc oxide nanoparticles (ZnO-NPs) induce salt tolerance by improving the antioxidant system and photosynthetic machinery in tomato. Plant Physiol. Biochem..

[8] Munns R., Tester M. (2008). Mechanisms of salinity tolerance. Annu. Rev. Plant Biol..

[9] Chrysargyris A., Michailidi E., Tzortzakis N. (2018). Physiological and biochemical responses of *Lavandula angustifolia* to salinity under mineral foliar application. Front. Plant Sci..

[10] He J., You X., Qin L. (2021). High salinity reduces plant growth and photosynthetic performance but enhances certain nutritional qualities of C_4_ halophy0te *Portullaca oleraceae* L. grown hydroponically under LED lighting. Front. Plant Sci..

[11] Lee J., Jeong J.S., Kim S.Y., Lee S.J., Shin Y.J., Im W.J., Kim S.H., Park K., Jeong E.J., Nam S.Y. (2020). Safety assessment of cerium oxide nanoparticles: Combined repeated-dose toxicity with reproductive/developmental toxicity screening and biodistribution in rats. Nanotoxicology.

[12] Rodea-Palomares I., Boltes K., Fernández-Pinas F., Leganés F., García-Calvo E., Santiago J., Rosal R. (2010). Physicochemical characterization and ecotoxicological assessment of CeO_2_ nanoparticles using two aquatic microorganisms. Toxicol. Sci..

[13] Park E.J., Choi J., Park Y.K., Park K. (2008). Oxidative stress induced by cerium oxide nanoparticles in cultured BEAS-2B cells. Toxicology.

[14] Srinivas A., Rao P.J., Selvam G., Murthy P.B., Reddy P.N. (2011). Acute inhalation toxicity of cerium oxide nanoparticles in rats. Toxicol Lett..

[15] Khorrami M.B., Sadeghnia H.R., Pasdar A.R., Ghayour-Mobarhan M., Riahi-Zanjani B., Hashemzadeh A.R., Zare M., Darroudi M. (2019). Antioxidant and toxicity studies of biosynthesized cerium oxide nanoparticles in rats. Int. J. Nanomed..

[16] Morales M.I., Rico C.M., Hernandez-Viezcas J.A., Nunez J.E., Barrios A.C., Tafoya A.T., Flores-Marges J.P., Peralta-Videa J.R., Gardea-Torresdey J.L. (2013). Toxicity assessment of cerium oxide nanoparticles in cilantro (*Coriandrum sativum* L.) plants grown in organic soil. J. Agric. Food Chem..

[17] Liu J., Li G., Chen L., Gu J., Wu H., Li Z. (2021). Cerium oxide nanoparticles improve cotton salt tolerance by enabling better ability to maintain cytosolic K^+^/Na^+^ ratio. J. Nanobiotechnol..

[18] Jahani S., Saadatmand S., Mahmoodzadeh H., Khavari-Nejad R.A. (2019). Effect of foliar application of cerium oxide nanoparticles on growth, photosynthetic pigments, electrolyte leakage, compatible osmolytes and antioxidant enzymes activities of *Calendula officinalis* L.. Biologia.

[19] Es-sbihi F.Z., Hazzoumi Z., Aasfar A., Amrani Joutei K. (2021). Improving salinity tolerance in *Salvia officinalis* L. by foliar application of salicylic acid. Chem. Biol. Technol. Agric..

[20] Khan M.I.R., Syeed S., Nazar R., Anjum N.A., Khan N.A., Nazar R., Iqbal N., Anjum N.A. An insight into the role of salicylic acid and jasmonic acid in salt stress tolerance. Phytohormones and Abiotic Stress Tolerance in Plants.

[21] Souri M.K., Tohidloo G. (2019). Effectiveness of different methods of salicylic acid application on growth characteristics of tomato seedling under salinity. Chem. Biol. Technol. Agric..

[22] El-Esawi M., Elansary H.O., El-Shanhorey N.A., Abdel-Hamid A.M.E., Ali H.M., Elshikh M.S. (2017). Salicylic acid-regulated antioxidant mechanisms and gene expression enhance rosemary performance under saline conditions. Fornt. Physiol..

[23] Abdoli S., Ghassemi-Golezani K., Alizadeh-Salteh S. (2020). Response of ajowan (*Trachyspormum ammi* L.) to exogenous salicylic acid and iron oxide nanoparticles under salt stress. Environ. Sci. Pollut. Res. Int..

[24] Zaman S., Hu S., Alam M.A., Du H., Che S. (2019). The accumulation of fatty acids in different organs of purslane under salt stress. Sci. Hortic..

[25] Alam M.A., Juraimi A.S., Rafii M.Y., Hamid A.A., Aslani F., Hasan M.M., Zainudin M.A.M., Uddin M.K. (2014). Evaluation of antioxidant compounds, antioxidant activities and mineral composition of 13 collected purslane (*Portulaca oleracea* L.) accessions. Biomed. Res. Int..

[26] Hasanuzzaman M., Borhaauddin Bhuyan M.H.M., Zulfiqar F., Raza A., Mohsin S.M., Al Mahmud J., Fujita M., Fotopoulos V. (2020). Reactive oxygen species and antioxidant defense in plants under abiotic stress: Revisiting the crucial role of a universal defense regulator. Antioxidants.

[27] Salehi H., Chehregani Rad A., Raza A., Chen J.T. (2021). 2021. Foliar application of CeO_2_ nanoparticles alters generative components fitness and seed productivity in Bean crop (*Phaseolus vulgaris* L.). Nanomaterials.

[28] Salehi H., Chehregani A., Lucini L., Majd A., Gholami M. (2018). Morphological Proteomic and metabolomic insight into the effect of cerium dioxide nanoparticles to *Phaseolus vulgaris* L. under soil or foliar application. Sci. Total Environ..

[29] Du W., Gardea-Torresdey J.L., Ji R., Yin Y., Zhu J., Peralta-Videa J.R., Guo H. (2015). Physiological and biochemical changes imposed by CeO_2_ nanoparticles nanoparticles on wheat: A life cycle field study. Environ. Sci. Technol..

[30] Jini D., Joseph B. (2017). Physiological mechanism of salicylic acid for alleviation of salt stress in rice. Rice Sci..

[31] Shaki F., Ebrahimzadeh Maboud H., Niknam V. (2017). Central role of salicylic acid in resistance of safflower (*Carthamus tinctorius* L.) against salinity. J. Plant Interact..

[32] Khan M.N., Li Y., Khan Z., Chen L., Liu J., Hu J., Wu H., Li Z. (2021). Nanocoria seed priming enhanced salt tolerance in rapeseed through modulating ROS homeostasis and α1-amylase activities. J. Nanobiotechnol..

[33] Singh S., Husen A., Husen A., Iqbal M. (2019). Role of nanomaterials in the mitigation of abiotic stress in plants. Nanomaterials & Plant Potential.

[34] Samadi S., Habibi G., Vaziri A. (2019). Effects of exogenous salicylic acid on antioxidative responses, phenolic metabolism and photochemical activity of strawberry under salt stress. Iran. J. Plant Physiol..

[35] Jurkow R., Sękara A., Pokluda R., Smoleń S., Kalisz A. (2020). Biochemical response of oakleaf lettuce seedlings to different concentrations of some metal (oid) oxide nanoparticles. Agronomy.

[36] Ahmed W., Imran M., Yaseen M., ul Hag T., Jamshaid M.U., Rukh S., Ikram R.M., Ali M., Ali A., Maqbool M. (2020). Role of salicylic acid in regulation ethylene and physiological characteristics for alleviating salinity stress on germination, growth and yield of sweet peppar. Peer J..

[37] Kim Y., Mun B.G., Khan A.L., Waqas M., Kim H.H., Shahzad R., Imran M., Yun B.W., Lee I.J. (2018). Regulation of reactive oxygen and nitrogen species by salicylic acid in rice plants under salinity stress conditions. PLoS ONE.

[38] Rossi L., Zhang W., Lombardini L., Ma X. (2016). The impact of cerium oxide nanoparticles on the salt stress responses of *Brassica napus* L.. Environ. Pollut..

[39] Kumar D., Al Hassan M., Naranjo M.A., Agrawal V., Boscaiu M., Vicente O. (2017). Effects of salinity and drought on growth, ionic relations, compatible solutes and activation of antioxidant systems in oleander (*Nerium oleander* L.). PLoS ONE.

[40] Torun H., Novak O., Mikulik J., Pencik A., Strnad M., Ayaz F.A. (2020). Timing-dependent effects of salicylic acid treatment on phytohormonal changes, ROS regulation, and antioxidant defense in salinized barley (*Hordeum vulgare* L.). Sci. Rep..

[41] Husen A., Iqbal M., Sohrab S.S., Ansari M.K.A. (2018). Salicylic acid alleviates salinity-caused damage to foliar functions, plant growth and antioxidant system in Ethiopian mustard (*Brassica carinata* A. Br.). Agric. Food Secur..

[42] Ma X., Zheng J., Zhang X., Hu Q., Qian R. (2017). Salicylic acid alleviates the adverse effects of salt stress on *Dianthus superbus* (Caryophyllaceae) by activating photosynthesis, protecting morphological structure, and enhancing the antioxidant system. Front. Plant Sci..

[43] Jangra M., Devi S., Satpal S.D., Kumar N., Goyal V., Mehrotra S. (2022). Amelioration effects of salicylic acid under salt stress in *Sorghum bicolor* L.. Appl. Biochem. Biotechnol..

[44] Kalisz A., Huska D., Jurkow R., Dvork M., Klejdus B., Caruso G., Sekara A. (2021). Nanoparticles of cerium oxide. Iron, and silicon oides change the metabolism of phenols and flavonoids in butterhead lettuce and sweet pepper seedling. Environ. Sci. Nano.

[45] Grzeszczuk M., Salachna P., Meller E. (2018). Change in photosynthetic pigments, total phenolic content and antioxidant activity of *Salvia coccinea* Buchoz Ex Etl. Induced by exogenous salicylic acid and soil salinity. Molecules.

[46] Sdouga D., Ben Amor F., Ghribi S., Kabtni S., Tebini M., Branca F., Trifi-Farah N., Marghali S. (2019). An insight from tolerance to salinity stress in halophyte *Portulaca oleracea* L.: Physio-morphological, biochemical and molecular responses. Ecotoxicol. Environ. Saf..

[47] Rossi L., Zhang W.L., Ma X.M. (2017). Cerium oxide nanoparticles alter the salt stress tolerance of *Brassica napus* L. by modifying the formation of root apoplastic barriers. Environ. Pollut..

[48] Wu H., Shabala L., Shabala S., Giraldo J.P. (2018). Hydroxyl radical scavenging by cerium oxide nanoparticles improves Arabidopsis salinity tolerance by enhancing leaf mesophyll potassium retention. Environ. Sci. Nano.

[49] Ahanger M.A., Tittal M., Mir R.A., Agarwal R.M. (2017). Alleviation of water and osmotic stress-induced changes in nitrogen metabolizing enzymes in *Triticum aestivum* L. cultivars by potassium. Protoplasma.

[50] Hassanpouraghdam M.B., Vojodi Mehrabani L., Badali R., Aazami M.A., Rasouli F., Kakaei K., Szczepanek M. (2022). Cerium oxide salicylic acid nanoparticles (CeO_2_: SA-NPs) foliar application and in-soil animal manure use influence the growth and physiological responses of *Aloe vera* L.. Agronomy.

[51] Prochazkova D., Sairam R.K., Srivastava G.C., Singh D.V. (2001). Oxidative stress and antioxidant activity as the basis of senescence in maize leaves. Plant Sci..

[52] Kim K.H., Tsao R., Yang R., Cui S.W. (2006). Phenolic acid profiles and antioxidant activities of wheat bran extracts and the effect of hydrolysis conditions. Food Chem..

[53] Wanger G.J. (1979). Contact and vacuole/extravacuole distribution of neutral sugars, free amino acids and anthocyanin in protoplast. Plant Physiol..

[54] Fedina I., Georgieva K., Velitchkova M., Grigorova I. (2006). Effect of pretreatment of barley seedlings with different salts on the level of UV-B induced and UV-B absorbing compounds. Environ. Exp. Bot..

[55] Alexieva V., Sergiev I., Mapelli S., Karanov E. (2001). The effect of drought and ultraviolet radiation on growth and stress markers in pea and wheat. Plant Cell Environ..

[56] Sahu G.K., Sabat S.C. (2011). Changes in growth, pigment content and antioxidants in the root and leaf tissues of wheat plants under the influence of exogenous salicylic acid. Braz. J. Plant Physiol..

[57] Luhova L., Lebeda A., Hederorva D., Pee P. (2003). Activities of oxidase, peroxidase and catalase in seedlings of *Pisum sativum* L. under different light conditions. Plant Soil Enviorn..

[58] Honarjoo N., Hajrasuliha S.H., Amini H. (2013). Comparing three plants in absorption of ions from different natural saline and sodic soils. Int. J. Agric. Crop Sci..

